# Cancer-associated fibroblasts-derived HAPLN1 promotes tumour invasion through extracellular matrix remodeling in gastric cancer

**DOI:** 10.1007/s10120-021-01259-5

**Published:** 2021-11-01

**Authors:** Tiancheng Zhang, Xiang Li, Yani He, Yaohui Wang, Jiajia Shen, Shoulin Wang, Qiang You, Jing Zhai, Lizong Shen

**Affiliations:** 1 Department of Surgical Oncology, Jiangsu Province Hospital of Chinese Medicine, Affiliated Hospital of Nanjing University of Chinese Medicine, 155 Hanzhong Road, Nanjing, 210029 China; 2 Digestive Endoscopy Center, Jiangsu Province Hospital of Chinese Medicine, Affiliated Hospital of Nanjing University of Chinese Medicine, Nanjing, 210029 China; 3 Department of Pathology, Jiangsu Province Hospital of Chinese Medicine, Affiliated Hospital of Nanjing University of Chinese Medicine, Nanjing, 210029 China; 4 Department of General Surgery, First Affiliated Hospital, Nanjing Medical University, Nanjing, 210029 China; 5 School of Public Health, Nanjing Medical University, Nanjing, 211166 China; 6 Department of Geriatrics, Second Affiliated Hospital, Nanjing Medical University, Nanjing, 210003 China

**Keywords:** Gastric cancer, Cancer-associated fibroblasts, HAPLN1, Tumour invasion, Extracellular matrix remodeling

## Abstract

**Background:**

Cancer-associated fibroblasts (CAFs) are the most principal cells of depositing and remodeling extracellular matrix (ECM) within solid tumours. Both CAFs and ECM have been demonstrated to play critical roles in tumour development. However, the functional roles of CAFs-associated ECM or ECM remodeling in the pathogenesis of gastric cancer remain unclear.

**Methods:**

Bioinformatics analysis of the differentially expressed genes between CAFs and corresponding normal fibroblasts (NFs) in gastric cancer was performed. The clinical relevance of hyaluronan and proteoglycan link protein 1 (HAPLN1) was investigated using TCGA data and human gastric cancer specimens. Spheroid cell invasion assay and nude mouse xenograft model were introduced to assay cell invasion. Second harmonic generation (SHG) was used to image and analyze the changes of collagen fibers in ECM.

**Results:**

HAPLN1 was identified as the most significantly up-regulated gene in CAFs of gastric cancer, and higher HAPLN1 levels were associated with shorter overall survival. HAPLN1 was prominently produced by CAFs, and its levels were correlated positively with tumor T staging (*P* < 0.0001), lymph node metastasis (*P* = 0.0006) and TNM stage (*P* = 0.0063). Mechanically, gastric cancer cells activate fibroblasts to up-regulate HAPLN1 expression via activation of TGF-β1/Smad2/3 signaling, which in turn promotes tumour migration and invasion. Importantly, SHG assays with mouse xenograft models and human samples further demonstrated CAFs-derived HAPLN1 increased tumour invasiveness through ECM remodeling.

**Conclusions:**

This study sheds light on the role of CAFs-derived HAPLN1 in the pathogenesis of gastric cancer, and provides insights for the development of novel strategies for prevention and treatment of gastric carcinoma.

**Supplementary Information:**

The online version contains supplementary material available at 10.1007/s10120-021-01259-5.

## Introduction

Gastric cancer remains one of the most common malignancies around the world with more than 1 million estimated new cases annually. Albeit a steady decline in the incidence observed in most settings over the past century, most cases are frequently diagnosed at advanced stages, making it the third most cause of cancer-associated deaths [1]. The tumour behaviors, especially invasion and metastasis, impose profoundly negative effects on the prognosis of cancer patients, and tumour biological characteristics are dictated not only by tumour parenchyma, but also by its mesenchyme.

Cancer-associated fibroblasts (CAFs) are the most prominent stromal cells in solid tumours including gastric cancer. CAFs act as a key regulator in cancer and exert diverse effects, including extracellular matrix (ECM) deposition and remodeling, extensive reciprocal signaling interactions with cancer cells and crosstalk with infiltrating myeloid cells [2, 3]. Our previous studies have demonstrated that CAFs-derived IL-8 mediates chemoresistance to cisplatin in gastric cancer via nuclear factor-κB (NF-κB) activation and up-regulation of ATP-binding cassette subfamily B member 1 (ABCB1) [4], and that CAFs-derived vascular adhesion molecule 1 (VCAM1) induced by *H. pylori* infection facilitates tumour invasion in gastric cancer via molecular interaction with integrin αvβ1/5 in tumour cells [5].

ECM is a non-cellular three-dimensional macromolecular network that provides the scaffold wherein tumour cells are located [6]. ECM is composed of about 300 unique matrix macromolecules, which can be classified into collagens, proteoglycans and glycoproteins [7]. These ECM molecules and their assembling manner determine the biochemical and biophysical properties of the resultant ECM [8]. ECM plays a critical role in tumour progression and dissemination, and dysregulated ECM remodeling by cancer cells facilitates cancer niche formation [9, 10]. CAFs serve as the most principal cell type of depositing and remodeling ECM within solid tumours [2, 11]. However, the roles of CAFs-associated ECM or ECM remodeling in the pathogenesis of gastric cancer are rarely documented.

In the present study, we performed bioinformatics analysis, and identified hyaluronan and proteoglycan link protein 1 (HAPLN1) as the most significantly up-regulated gene in CAFs compared with the respective normal fibroblasts (NFs) of human gastric cancer. Given that HAPLN1 is the main ECM-modifying protein [7], we further identified that HAPLN1 is mainly produced by CAFs in human gastric cancer microenvironment, which is associated with the disease progression and poor prognosis. Gastric cancer cells can activate fibroblasts to up-regulate HAPLN1 expression via activation of TGF-β1/Smad2/3 signaling. Furthermore, using the second harmonic generation (SHG) imaging with a multi-photon microscope, we demonstrated that CAFs-derived HAPLN1 promotes gastric cancer invasion through the ECM remodeling, including decreasing the number, density, width and length of fibers, as well as increasing the fiber alignment. Our studies shed a light on the role of CAFs-derived HAPLN1 in the pathogenesis of gastric cancer, and should provide novel CAFs-based strategies for the prevention and treatment of gastric carcinoma.

## Materials and methods

Multiple materials and methods see the Supplementary materials and methods.

### Human serum and tissue specimens of gastric cancer patients

This study enrolled 155 patients with primary gastric carcinoma from March 2018 to June 2020 at the Department of Surgical Oncology, Affiliated Hospital of Nanjing University of Chinese Medicine. All these patients were pathologically diagnosed as stomach adenocarcinoma following the American Joint Committee on Cancer (AJCC) criteria. All these patients had not received preoperative chemotherapy or radiotherapy. The serum specimens from patients were collected preoperatively. The tumour tissues and the corresponding noncancerous mucosa tissues (at least 5 cm from the outer tumour margin) were collected from all patients immediately after resection and snap frozen in liquid nitrogen. Isolation and culture of primary gastric cancer CAFs and primary stomach NFs in corresponding non-cancerous tissues were performed according to our previous report [4, 5] and referring the newly published protocol of Yasuda et al. [12]. The samples were obtained following written consent according to an established protocol approved by the Institutional Review Board of Nanjing University of Chinese Medicine. This study was also in accordance with the Declaration of Helsinki.

### Bioinformatics analysis

The RNA-seq data sets were downloaded from Gene-Expression Omnibus (GEO, https://www.ncbi.nlm.nih.gov/geo/), including six pairs of primary CAFs and the respective primary NFs isolated from advanced gastric cancer tissues (accession code GSE83834). Difference analysis was performed between CAFs and NFs mRNA expression data using the R packaged “edgeR 3.30.3”. Genes with |log2FoldChange|> 1 and FDR < 0.05 was considered differentially expressed. The R package “ggplot2 3.3.0” was used for volcano plot and the R package “ComplexHeatmap 2.4.3” was used to draw the heat map.

### Immunohistochemistry (IHC)

IHC assays were conducted according to the standard protocols on 4-mm paraffin sections of formalin-fixed, paraffin-embedded resected stomach specimens. Rabbit monoclonal anti-HAPLN1 and anti-TGF-β1 antibodies (Abcam, Cambridge, UK) were used. The staining intensity of tumor cells and the percentage of positive cells were scored by two pathologists. Staining intensity: 0 for no staining, 1 for weak staining, 2 for moderate staining and 3 for strong staining. The percentage of positive cells: 1 for ≤ 10% positive cells, 2 for 1–50% positive cells, 3 for 51–75% positive cells and 4 for > 75% positive cells. If the multiply of the two scores > 3, positive IHC expression was defined.

### Chromatin immunoprecipitation (ChIP)

ChIP assays were performed with the SimpleChIP ^®^Enzymatic Chromatin IP Kit (Cell Signaling Technology, MA, USA) following manufacturer’s recommended protocols. Cell lysates of CAFs (4 × 10^7^) were prepared, and chromatin fragments were fragmented to an average size of 150–900 bp by microcapsule nuclease, and enriched with magnetic beads coated with Smad2/3 antibody, isotype IgG and Histone H3. Then, the concentrated sample was crosslinked with the input DNA, and the DNA was purified with sodium chloride and protease K. Finally, the specific sequences from immunoprecipitated and input DNA were determined by real-time quantitative PCR (qPCR) for the upstream of HAPLN1 promoter region. Three primer pairs of HAPLN1 promoter region used in qPCR analyses were listed in Table s1, and the pair #3 was used in this assay.

### RNA interference analysis

Lentivirus carrying HAPLN1 short hairpin RNA (shRNA) was constructed by Shanghai GenePharma Co., Ltd. (Shanghai, China). The transduction was performed according to the manufacturer’s recommended protocols. The shRNA targeting sequences for HAPLN1 were listed in Table s2. The knockdown efficiency was verified by western blotting assay, and shRNA #1 was selected.

### Establishment of subcutaneous xenograft tumour model of gastric cancer in Balb/c nude mouse

Animal studies were approved by the Animal Management and Use Committee of Nanjing University of Chinese Medicine. Balb/c nu/nu mice (SPF, 4–6 weeks) were obtained from the Institute of Biomedical Sciences, Nanjing University. These mice were randomly divided into four groups, and each contained five mice. After digestion and washing, MKN45 cells and CAFs cells with different HAPLN1 levels were mixed in a ratio of 1 to 1, and were inoculated into the hypochondrium of mice subcutaneously. Recombinant HAPLN1 (rHAPLN1) (Sino Biological, Beijing, China) in the indicated group was administrated with cell mixture simultaneously (25 μg/mouse). Four weeks later, these mice were euthanized and these tumours were obtained. Tumour volume was calculated using width × length × (width + length)/2.

### Second harmonic generation (SHG) imaging of collagen fibers with a multi-photon microscope

Collagen fibers were imaged using a laser scanning confocal microscope LSM880 (Carl Zeiss, Germany) with SHG generated at 450 nm. After imaging, the characteristics of collagen fibers were assessed, including number, density, length, width, straightness and alignment. Fiber density was detected by ImageJ, and the others were analyzed by CT-FIRE (version number: v1.3 Beta2) and Matlab compiler MCR 7.17 2012a.

### Statistical analysis

GraphPad software (Prism 8.0) was used for statistical analysis. The *t* test was used to compare continuous variables between the two groups, and the Chi-square test was used to compare categorical variables. The overall survival was determined by Kaplan–Meier and Gehan-Breslow-Wilcoxon tests. All experiments were repeated at least three times. All values in the text and graph deviate from the mean standard. *P* less than 0.05 was considered significant.

## Results

### HAPLN1, differentially expressed in CAFs, is associated with the disease progression and poor prognosis in gastric cancer

**Fig. 1:**
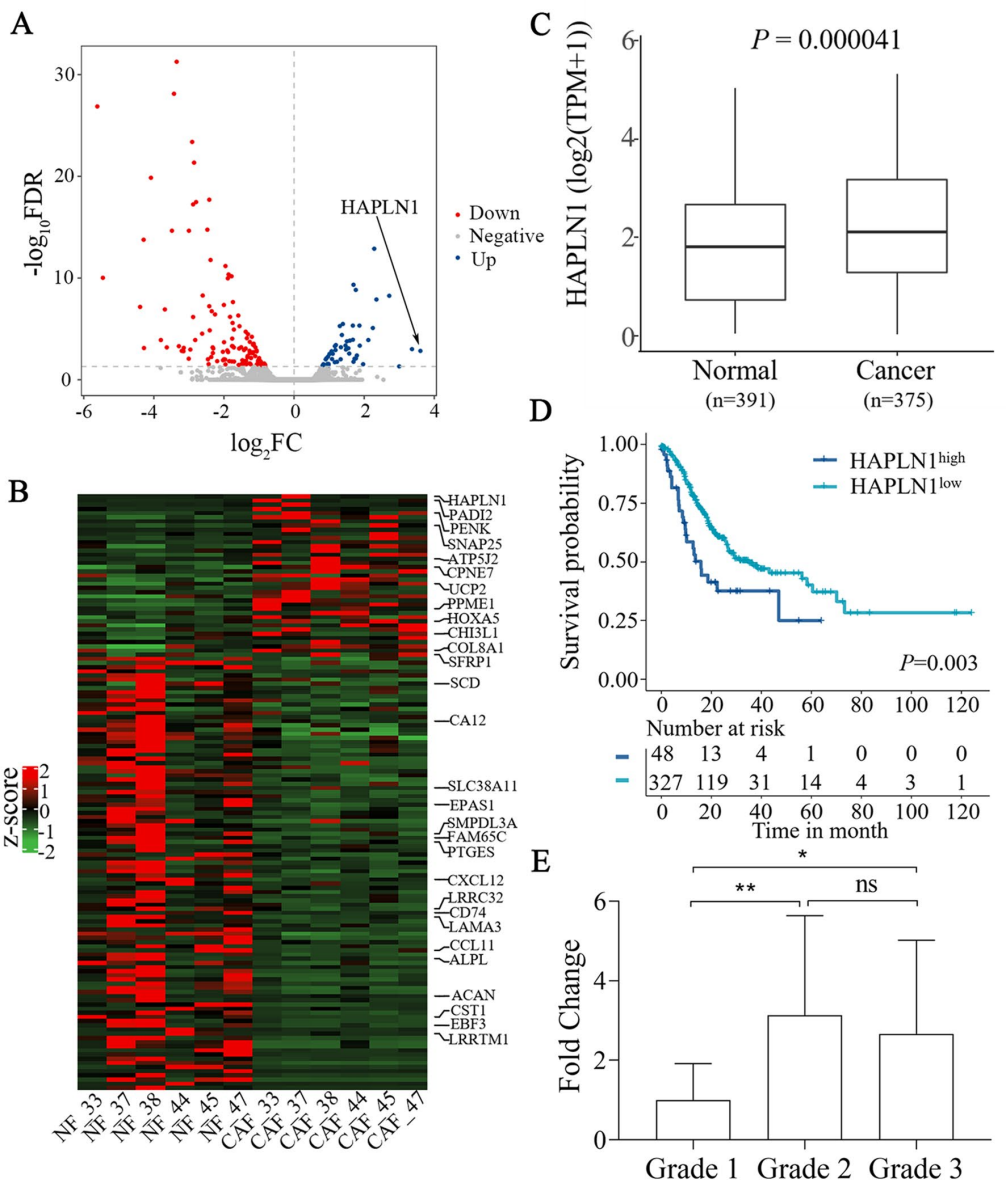
HAPLN1 is highly expressed in cancer-associated fibroblasts (CAFs), and is associated with the disease progression and poor prognosis in gastric cancer. **A** Bioinformatics analysis of the differentially expressed genes (DEGs) between CAFs and corresponding NFs of human gastric cancer indicated HAPLN1 was highly expressed in CAFs (*P* = 7.81 × 10^–6^). **B** The heat map of DEGs between CAFs and NFs. **C** Higher levels of HAPLN1 mRNA were detected in the cancer tissues of TCGA data with regard to the normal stomach tissues (*P* = 0.000041). **D** Higher HAPLN1 levels were associated with shorter overall survivals (OS) in gastric cancer patients of TCGA data (*P* = 0.003). **E** HAPLN1 mRNA level was higher in the moderately (Grade 2) or poorly (Grade 3) differentiated cancers than that in the well differentiated cancers (Grade 1) detected by qPCR (*P < 0.05, **P < 0.01)

To dissect the role of CAFs in the pathogenesis of gastric cancer, we first performed a profound bioinformatics analysis of the differentially expressed genes (DEGs) in CAFs compared with the corresponding NFs of human gastric cancer using the data in the GEO database (accession code GSE83834) [13]. We found that HAPLN1 was the most differentially up-regulated gene in the CAFs compared with that in NFs (*P* = 7.81 × 10^–6^) (Fig. 1A, B). To probe the clinical significance of HAPLN1 in gastric cancer, we analyzed the correlations between HAPLN1 expression and clinical outcomes using the clinical data of gastric cancer patients from the Cancer Genome Atlas (TCGA) and GTEx (Genotype-Tissue Expression) databases (https://gtexportal.org/home). High levels of HAPLN1 mRNA were detected in the cancer tissues compared with the normal stomach tissues (Fig. 1C, P = 0.000041). The Kaplan–Meier analysis indicated that higher HAPLN1 level was associated with a shorter overall survival (OS) in gastric cancer patients (Fig. 1D, P = 0.003). Our retrospective study revealed HAPLN1 mRNA level was higher in the moderately (Grade 2) (*P* < 0.01) or poorly (Grade 3) (*P* < 0.05) differentiated cancers than that in the well differentiated cancers (Grade 1) detected by qPCR (Fig. 1E). The IHC assays further showed that high HAPLN1 levels were observed in 51.61% patients with locally advanced gastric cancer (Table s3), and its levels were positively correlated with tumour T staging (*P* < 0.0001), lymph node metastasis (LNM) (*P* = 0.0006) and TNM stage (*P* = 0.0063). Collectively, HAPLN1 expression in gastric cancer is associated with the disease progression and poor outcome.

### HAPLN1 is mainly produced by CAFs in gastric cancer

**Fig. 2:**
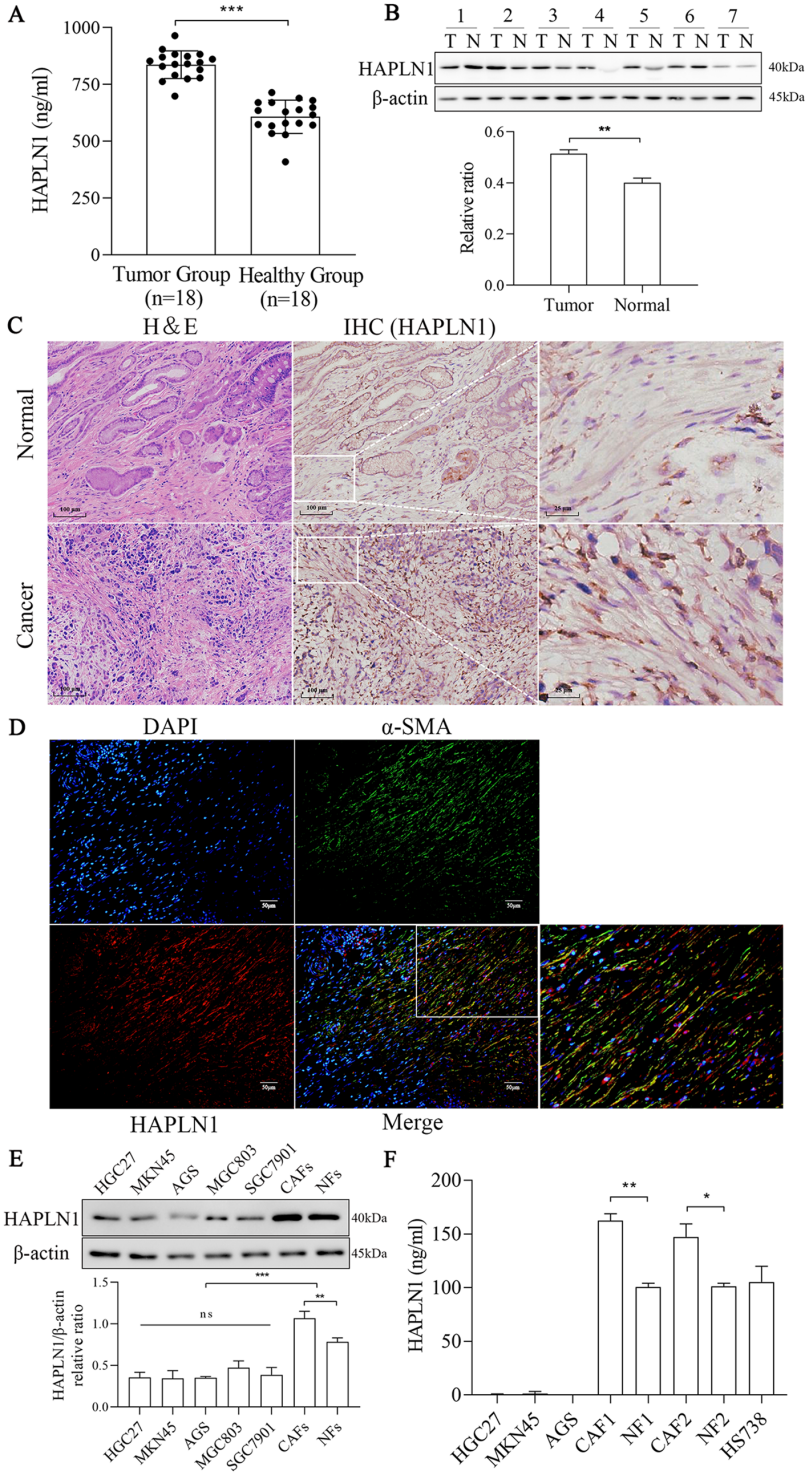
HAPLN1 is derived from CAFs in gastric cancer. **A** The serum HAPLN1 levels in gastric cancer patients were higher than that in healthy controls. **B** HAPLN1 was up-regulated in gastric cancer tissues than that in normal stomach tissues. **C** HAPLN1 was expressed prominently in CAFs in cancer tissues and in NFs in normal tissues, and its level was higher in CAFs than that in NFs. HAPLN1 expression in the indicated frames was amplified, respectively. **D** Immunofluorescence assay approved that HAPLN1 was mainly located in CAFs, and the right lower one was amplification of the white frame to showing the co-expression of α-SMA and HAPLN1. **E** Western blotting assay indicated that HAPLN1 was mainly expressed in fibroblasts (primary CAFs and primary NFs) rather than in gastric cancer cell lines (HGC27, MKN45, AGS, MGC803 and SGC7901). **F** ELISA assays indicated HAPLN1 was mainly detected in the supernatant of primary CAFs (CAF1, CAF2), respective primary NFs (NF1, NF2) and human stomach fibroblast line Hs738 rather than in the supernatant of tumour cells (HGC27, MKN45 and AGS). CAFs produced more HAPLN1 than the corresponding NFs. (*P < 0.05, **P < 0.01, ***P < 0.001)

We then performed the validation studies to further confirm that the serum HAPLN1 levels in gastric cancer patients were much higher than those in healthy controls (836.4 ± 60.88 ng/ml *vs* 606.8 ± 73.37 ng/ml, *P* < 0.001) (Fig. 2A). Furthermore, HAPLN1 expression was up-regulated in cancer tissues compared with normal stomach tissues (Fig. 2B, P < 0.01). To further dissect the origin of HAPLN1 in the tumour microenvironment, we demonstrated that HAPLN1 was expressed prominently in CAFs in cancer tissues and in NFs in normal tissue as well. However, the expression level of HAPLN1 was much higher in CAFs than that in NFs (Fig. 2C). The immunofluorescence assay confirmed that HAPLN1 was mainly located in CAFs (Fig. 2D). Studies using the human gastric cancer cell lines and the primary gastric cancer CAFs or NFs revealed that HAPLN1 was mainly expressed in the fibroblasts rather than in cancer cells of HGC27, MKN45, AGS, MGC803 and SGC7901 cells. Furthermore, CAFs produced more HAPLN1 than that of the corresponding NFs (Fig. 2E, F). These findings suggest that HAPLN1 is mainly derived from CAFs in gastric cancer.

### Gastric cancer cells promote HAPLN1 production by CAFs via the activation of TGF-β1/Smad signaling

**Fig. 3:**
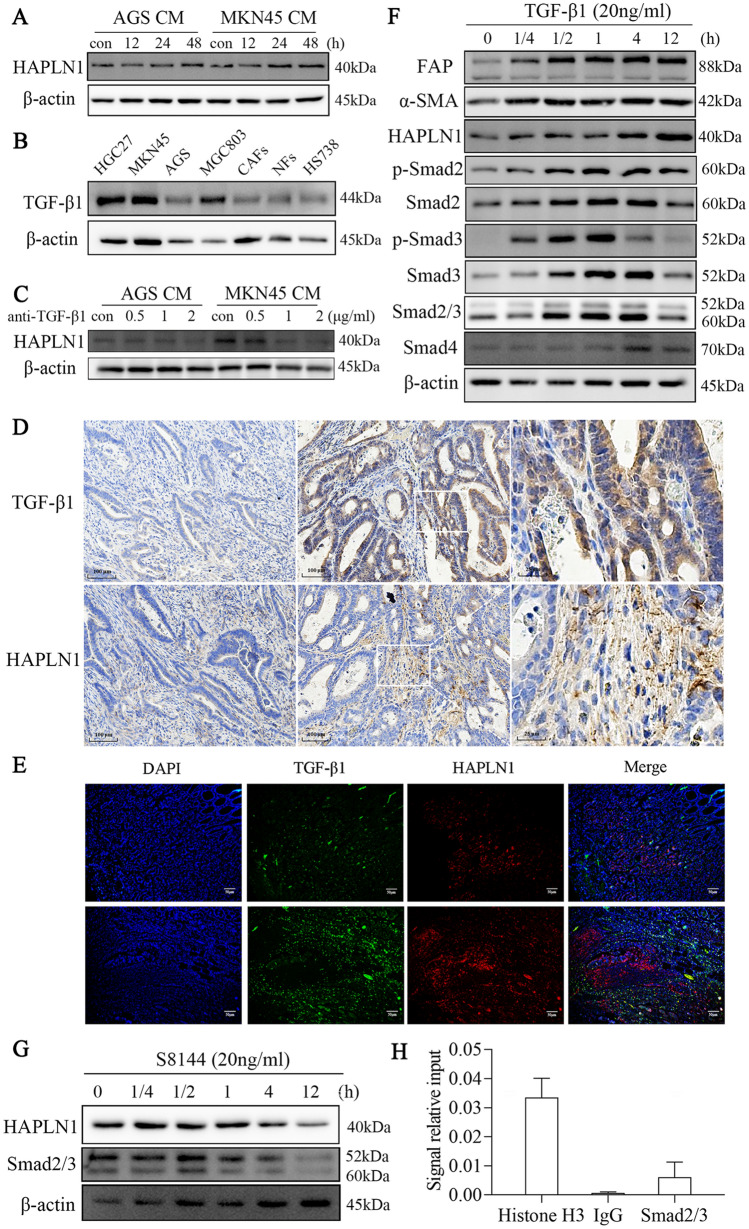
Gastric cancer cells promote HAPLN1 production in CAFs via activation of TGF-β1/Smad signaling. **A** Treatment with the condition medium (CM) of AGS cells or MKN45 cells increased HAPLN1 expression of Hs738 cells in a time-dependent manner. **B** TGF-β1 was expressed in human gastric cancer cell lines, including HGC27, MKN45, AGS and MGC803 cells, and was seldom detected in primary CAFs and NFs, as well as human stomach fibroblast line Hs738 cell. **C** Anti-TGF-β1 neutralizing monoclonal antibody could redeem the effects of AGS CM or MKN45 CM on HAPLN1 expression. **D** IHC assays with human tumour samples showed that TGF-β1 expression in cancer cells was consistent with HAPLN1 expression in CAFs. HAPLN1 or TGF-β1 expression in the indicated frames was amplified, respectively. **E** Immunofluorescence assays further showed that TGF-β1 in tumour cells and HAPLN1 in CAFs were detected simultaneously in gastric cancer tissues. **F** TGF-β1 up-regulated the expression of FAP and α-SMA in a time-dependent manner, and HAPLN1, Smad2, Smad3, p-Smad2, p-Smad3, Smad2/3 and Smad4 were up-regulated concurrently. **G** S8144, a Smad3 inhibitor, could down-regulated both HAPLN1 and Smad2/3 expression in Hs738 cells in a time-dependent manner. **H** ChIP-qPCR assays revealed that HAPLN1 DNA binding with Smad2/3 was much higher than IgG

To dissect the mechanism underlying HAPLN1 up-regulation in CAFs, we first treated human stomach fibroblast cell line Hs738 with the condition medium (CM) of human gastric cancer cell lines, AGS cells and MKN45 cells. We found that the HAPLN1 expression in Hs738 cells was significantly increased in a time-dependent manner after treatment with tumour CM (Figs. 3A, s1A). TGF-β1 has been recognized as one of the key factors of tumour cells that converts normal fibroblasts into CAFs [14]. We showed that TGF-β1 was expressed in human gastric cancer cell lines, including HGC27, MKN45, AGS and MGC803 cells, but was seldom detected in the primary CAFs, as well as in NFs and Hs738 cell line (Figs. 3B, s1B). Importantly, neutralization of TGF-β1 activity with an anti-TGF-β1 monoclonal antibody (Biolegend, San Diego, USA) could prevent the increase of HAPLN1 expression and production in Hs738 cells induced by gastric cancer cell CM (Figs. 3C, s1C, s1D). IHC and immunofluorescence assays with human tumour samples showed that TGF-β1 expression in cancer cells was correlated with HAPLN1 expression in CAFs (Fig. 3D, E and Table s4). These results indicate that gastric cancer cells up-regulate HAPLN1 in CAFs via TGF-β1.

To further explore the mechanisms responsible for TGF-β1-induced promotion of HAPLN1 expression in CAFs, we treated Hs738 cells with recombinant TGF-β1. We found that TGF-β1 treatment can up-regulate the expression of FAP and α-SMA in Hs738 cells with a time-dependent manner. Therese results suggest that TGF-β1 activates human stomach fibroblasts, and HAPLN1 could be a potential marker of the activated fibroblasts. To explore the molecular processes of TGF-β1 signaling in regulation of HAPLN1 expression in CAFs, we demonstrated that Smad2, Smad3, phosphorylated Smad2 (p-Smad2), p-Smad3 and Smad4 were up-regulated concurrently (Figs. 3F, s1E). Importantly, Smad2/3 expression also increased. Treatment with gastric cancer cell CM also increased Smad2/3 expression in Hs738 cells (Fig. s1F), while neutralization with anti-TGF-β1 inhibited these effects accordingly (Fig. s1G). Furthermore, treatment with S8144 (Selleck, Houston, USA), a Smad3 inhibitor, could down-regulate both HAPLN1 and Smad2/3 expression in Hs738 cells in a time-dependent manner (Figs. 3G, s1H). In addition, ChIP-qPCR assays revealed that HAPLN1 DNA binding with Smad2/3 was much higher than that with IgG, indicating that Smad2/3 is the transcriptional factor controlling HAPLN1 expression (Fig. 3H). These results collectively suggest that gastric cancer cells can activate fibroblasts and up-regulate the expression of HAPLN1 via the TGF-β1/Smad2/3 signaling activation.

### CAFs-derived HAPLN1 promotes gastric cancer cell invasion and migration in vitro

**Fig. 4:**
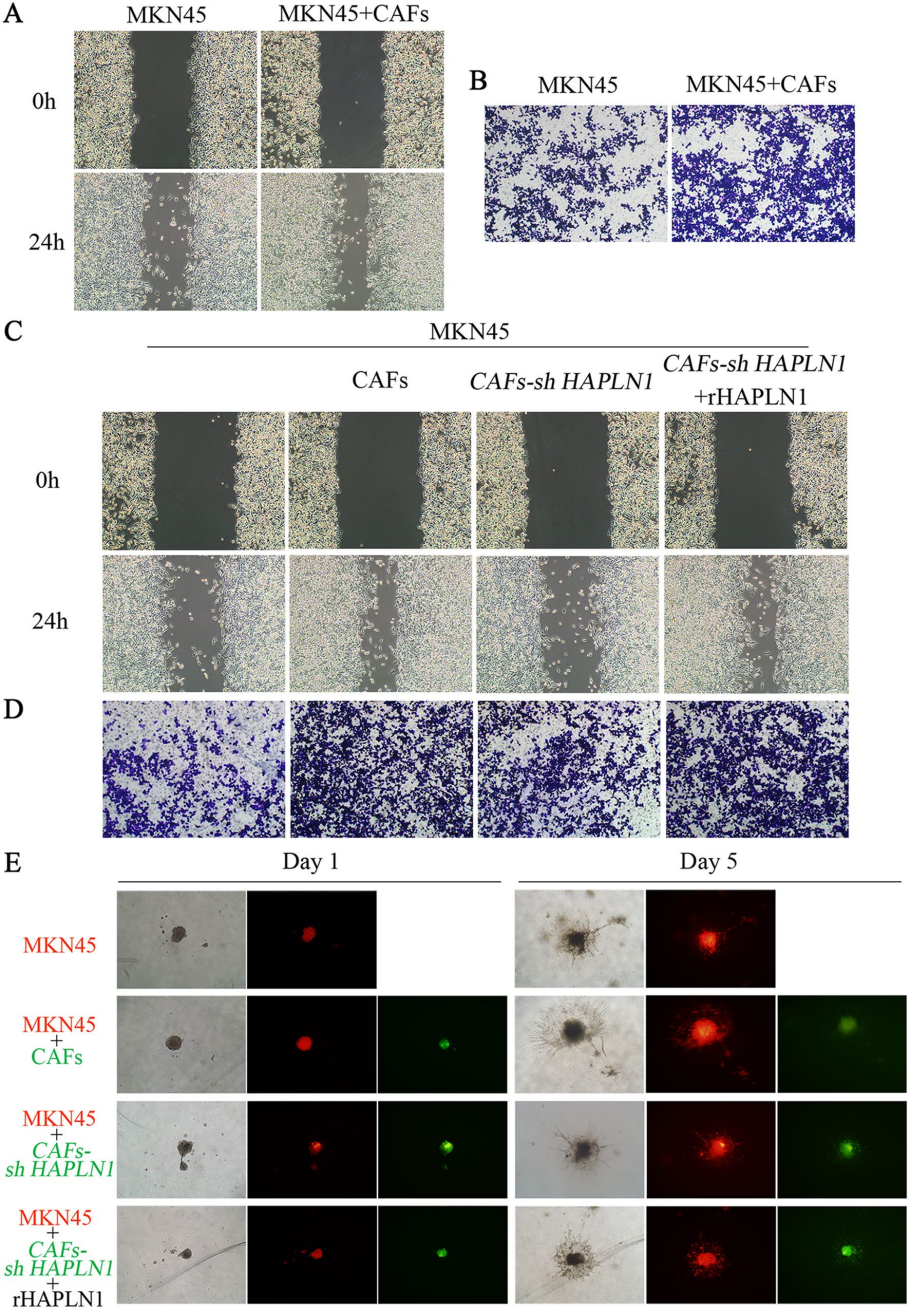
CAFs-derived HAPLN1 promotes gastric cancer invasion and migration. **A** Wound-healing assay showed that CAFs supernatant promoted cell migration of MKN45. **B** Transwell assay indicated that CAFs supernatant promoted MKN45 cell invasion. **C** and **D** CAFs supernatant promoted cell migration and invasion of MKN45; however, treatment with *CAFs-sh HAPLN1* supernatant inhibited this effects. MKN45 cell migration or invasion was recovered by treatment with recombinant HAPLN1 (rHAPLN1). **E** 3D spheroid cell invasion assay showed that CAFs increased MKN45 cell invasion, while HAPLN1 knockdown dramatically reduced the CAFs-mediated promotion of invasive ability of MKN45 cells. Addition of rHAPLN1 could recover these effects

We next investigated the effects of HAPLN1 on biological functions of gastric cancer cells. The wound-healing and Transwell assays showed the supernatant of CAFs significantly promoted migration (*P* < 0.01) and invasion (*P* < 0.05) of MKN45 cells (Figs. 4A, B, s2A, s2B). To functionally confirm the regulation mediated by HAPLN1, we designed and constructed lentivirus-based shRNAs against HAPLN1, and then generated a primary CAF cell line with knocked down HAPLN1 expression (*CAFs-sh HAPLN1*) (Fig. s2C). Intriguingly, treatment with supernatant of the *CAFs-sh HAPLN1* cells could significantly decrease the invasion and migration of MKN45 cells mediated by corresponding CAFs supernatant (*P* < 0.05). Importantly, the decreased MKN45 cell invasion and migration by *CAFs-sh HAPLN1* supernatant were recovered by co-culture with rHAPLN1 (*P* < 0.05) (Figs. 4C, D, s2D, s2E). We further performed 3D spheroid cell invasion assay using MKN45 cells and CAFs with different HAPLN1 levels. We showed that CAFs increased MKN45 cell invasion (*P* < 0.05), and that HAPLN1 knockdown dramatically reduced the CAFs-mediated promotion of invasiveness of MKN45 cells (*P* < 0.05), which could be recovered by addition of rHAPLN1 (*P* < 0.05) (Figs. 4E, s2F, s2G). These results collectively indicate that CAFs-derived HAPLN1 could promote gastric cancer cell migration and invasion.

### CAFs-derived HAPLN1 promotes gastric cancer cell tumourigenesis in nude mice through the extracellular matrix remodeling

**Fig. 5:**
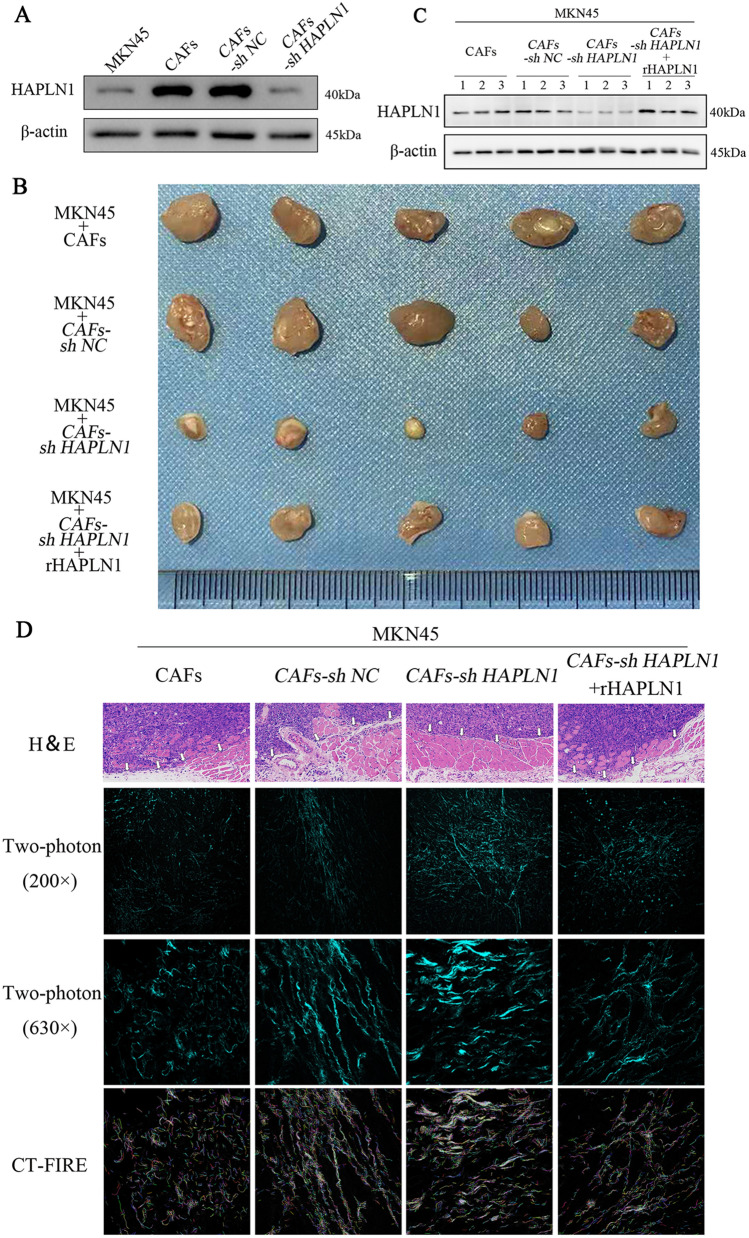
CAFs-derived HAPLN1 promotes gastric cancer tumourigenesis via ECM remodeling. **A** HAPLN1 expression in CAFs was inhibited by shRNA. **B** Nude mice xenograft model studies showed that the tumour volume in MKN45/*CAFs-sh HAPLN1* group was smaller than that in MKN45/CAFs group, and rHAPLN1 counterbalanced this effect significantly. **C** Assays with these xenograft tumours in mice confirmed the HAPLN1 levels in different groups. **D** Pathological studies revealed that tumour cells invaded the striated muscle tissues to different extent in the different groups (blank arrows: invasive frontier). Two-photon microscope assays showed that HAPLN1 knockdown increased the number, density, width and length of fibers in ECM, and reduced the fiber alignment significantly, while rHAPLN1 treatment could counteract these effects of HAPLN1 knockdown on ECM remarkably. (sh NC: null control of shRNA)

We further probed the effects of CAFs-derived HAPLN1 on gastric cell tumourigenesis in vivo. MKN45 cells and CAFs with different HAPLN1 levels were inoculated into Balb/c nu/nu mice subcutaneously and established xenograft tumours (Figs. 5A, s3A). Nude mouse studies indicated that the tumour volume in MKN45/*CAFs-sh HAPLN1* group was smaller than that in MKN45/CAFs group (Figs. 5B, s3B, *P* < 0.001), indicating that HAPLN1 knockdown in CAFs can inhibit MKN45 tumourigenesis in vivo. As expected, rHAPLN1 counterbalanced this effect significantly (*P* < 0.01). We also confirmed the HAPLN1 levels in isolated tumour tissues from different groups (Figs. 5C, s3C). Pathological studies revealed that tumour cells invaded the striated muscle tissues (Fig. 5D). However, HAPLN1 silence in CAFs inhibited MKN45 cell invasion, while rHAPLN1 addition recovered MKN45 cell invasiveness, although the extent was not appropriate for quantitative analysis. To elucidate the underlying mechanisms, we investigated the changes of ECM among these tumours using a two-photon microscope, and the data were analyzed with CT-FIRE. We showed that HAPLN1 knockdown increased the number, density, width and length of fibers in the ECM, but reduced the fiber alignment significantly. In contrast, rHAPLN1 treatment could counteract these effects induced by HAPLN1 knockdown on ECM remarkably (Figs. 5D, s3D). These findings implicate that CAFs-derived HAPLN1 leads to ECM remodeling, and thus promotes tumour invasion.

### HAPLN1 levels in CAFs are associated with tumour differentiation and extracellular matrix remodeling in human gastric cancer

**Fig. 6:**
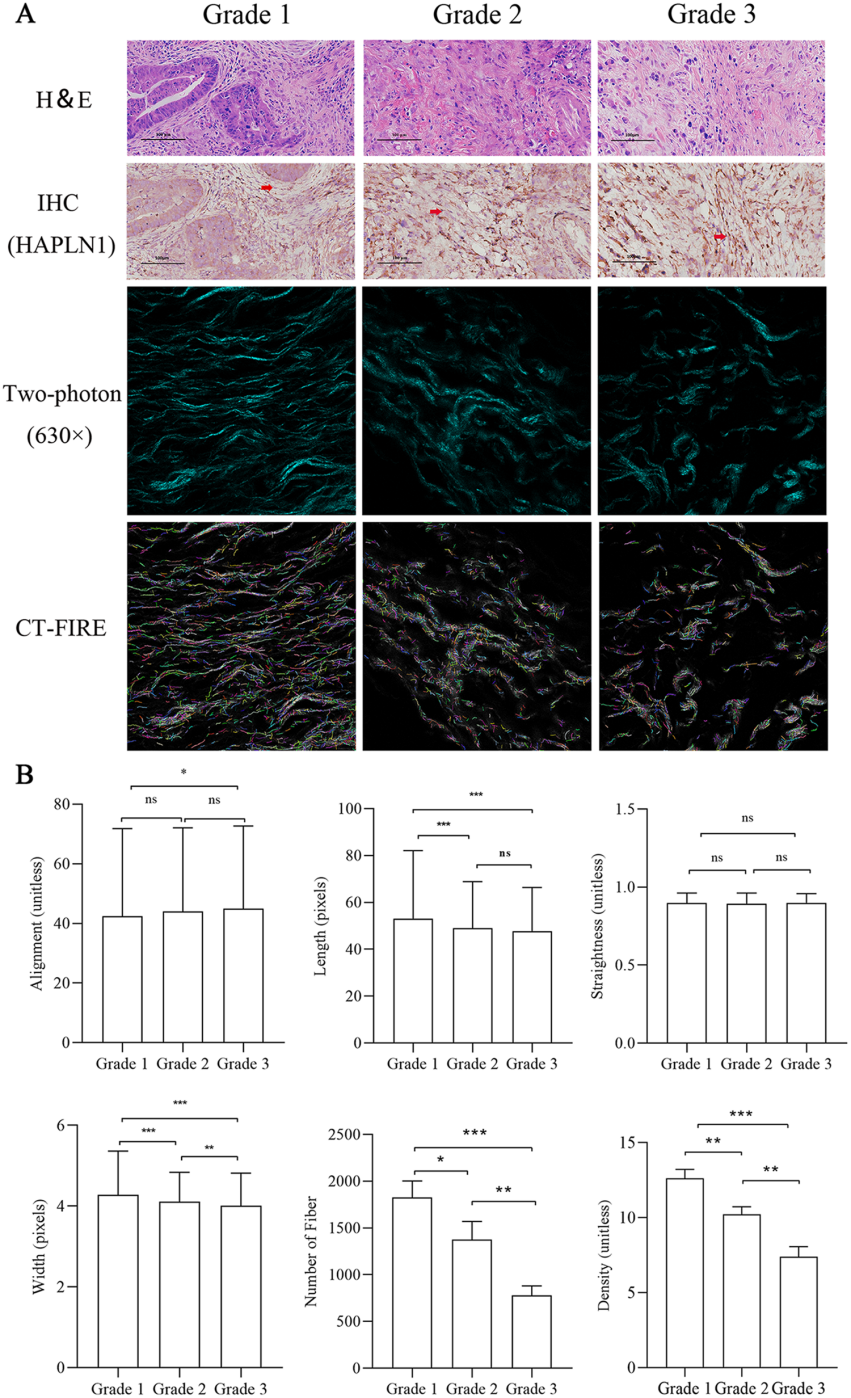
CAFs-derived HAPLN1 was associated with ECM remodeling in human gastric cancer. **A** IHC studies showed that HAPLN1 expression (red arrow) was increased with Grade 1 to Grade 3 significantly in gastric cancer. Two-photon assays showed that the number, density, width and length of fibers in ECM were decreased from Grade 1 to Grade 3 significantly, while the fiber alignment was increased (**B**). (*P < 0.05, **P < 0.01, ***P < 0.001)

We further investigated the causative association between HAPLN1 expression with tumour differentiation and ECM in human gastric cancer. Fifteen gastric carcinoma patients with different tumour differentiation, including five cases of Grade 1, five cases of Grade 2 and five cases of Grade 3, were enrolled. Hematoxylin–eosin (H & E) staining was performed to define the grades of tumour differentiation. As expected, we found that stromal HAPLN1 expression levels were increased with Grade 1 to Grade 3 significantly (Figs. 6A, s3E). SHG assay and CT-FIRE analysis showed that the number, density, width and length of fibers in ECM were decreased from Grade 1 to Grade 3 significantly, but the fiber alignment was increased (Fig. 6B), which was consistent with the findings shown in above mentioned animal studies. Collectively, the studies of human tumour tissues further confirm that CAFs-derived HAPLN1 is a key factor that drives tumour invasion through ECM remodeling.

## Discussion

CAFs have been demonstrated to play critical roles in the tumourgenesis, progression and dissemination of solid cancers, including gastric cancer [3, 5]. Furthermore, CAFs are one of the most effective cell types at depositing and remodeling the ECM to support tumour growth [2, 15]. In the present study, we identify HAPLN1 as the most significantly up-regulated molecule in CAFs compared with the corresponding NFs of gastric cancer. We further reveal that gastric cancer cells can activate fibroblasts to up-regulate HAPLN1 expression via the TGF-β1/Smad2/3 signaling activation, and that CAFs-derived HAPLN1 promotes tumour invasion through ECM remodeling.

ECM plays a crucial role in cell proliferation, differentiation and maintenance of tissue homeostasis [16]. However, the delicate regulation of ECM is disturbed in cancer, and dysregulated ECM remodeling generates molecular cues for cancer formation and progression [9]. Alteration of ECM stiffness and architecture makes the tumour microenvironment (TME) a mechanically complex niche, which reprograms cancer cell phenotype and primes cell invasion [10]. As the most prominent ECM-modifying protein, HAPLN1 is produced by fibroblasts [17–19]. Our bioinformatics analysis of DEGs between CAFs and NFs, as well as the validation studies have implicated that HAPLN1 is mainly derived from CAFs in gastric cancer.

Limited information is known about the clinical relevance of HAPLN1 with the disease progressions, including cancer. Long et al. identified ECM components HAPLN1, lumican and collagen I (HLC) induce human neocortex folding, which involves increase of ECM stiffness and HA/CD168/ERK signaling pathway. Disruption of this mechanism perturbs the physiological folding of human neocortical tissue [20]. Huynh et al. reported that HAPLN1 is produced by bone marrow stromal cells in multiple myeloma (MM) patients and it can activate an atypical bortezomib-resistant NF-κB pathway in MM cells. Thereby, HAPLN1 is proposed as a novel pathogenic factor to promote bortezomib resistance in MM cells [18]. We herein demonstrated that HAPLN1 expression in gastric cancer tissues was increased with relative to the normal stomach tissues. HAPLN1 could be detected in gastric cancer patients’ sera, and its serum levels in patients were higher than those in the healthy controls. HAPLN1 mRNA levels in tumour tissues were positively associated with the T staging, LNM and TNM stage in gastric cancer. Importantly, higher HAPLN1 levels were correlated with poorer overall survivals. The current study is the first report, to our knowledge, to investigate HAPLN1’s clinical significance in gastrointestinal cancer. However, Ecker et al. observed a threshold effect of improved overall survival associated with the upper quartile of HAPLN1 expression in melanoma [21], while Ivanova et al. found high expression of HAPLN1 was negatively correlated with time to progression and overall survival in malignant pleural mesothelioma [22]. These inconsistent effects of HAPLN1 on the prognosis of patients with different tumours suggested that roles of fibroblasts or HAPLN1 might be tissue-dependent [23–25]. Regarding the regulation of HAPLN1 expression, Chen et al. showed that Metformin, an AMP-activated protein kinase (AMPK) activator, promotes HAPLN1 secretion in rheumatoid arthritis (RA)-fibroblast-like synoviocytes (FLSs), but it inhibits FLSs activation [19]. We identified herein that gastric cancer cell-derived TGF-β1 up-regulates HAPLN1 expression in stomach fibroblasts through TGF-β1/Smad signaling activation.

HAPLN1 is a cross-linking protein, and it stabilizes proteoglycan monomer by aggregating with hyaluronan (HA) [26, 27]. HA is a structurally simple polysaccharide, and HA-rich microenvironments are conducive for tumour cells to initiate, survive, progress, escape therapeutic intervention and disseminate [28]. HA alterations have been shown to increase the ability of fibroblasts to contract collagen matrices, suggesting that changes in HAPLN1 could affect collagen cross-linking and ECM contractility [17]. We focused on the function of HAPLN1, and found that HAPLN1 promoted gastric cancer cell migration and invasion in vitro and in vivo. Importantly, we introduced second harmonic generation imaging, a highly specific technology for detection of collagen fibers, to analyze the change of ECM induced by HAPLN1. Both the mouse xenograft model study and human gastric cancer tissue assays indicated that HAPLN1 can reduce the number, density, width and length of collagen fibers in ECM, and increase the fiber alignment. We proposed that these changes of ECM are associated with increase of tumour invasiveness induced by HAPLN1. Winkler et al. summarized that the remodeling mechanisms of ECM include ECM deposition, post-translational chemical modification, proteolytic degradation and force-mediated physical remodeling [7]. Zhou et al. reported that the alignment, density, width, length and straightness of collagen fibers in gastric cancer microenvironment increase significantly with relative to normal stomach tissues, and showed that increased collagen width is associated with reduced survival [29]. However, we demonstrated that the reduced collagen density, width and length, as well as increased collagen alignment were involved in increased tumour invasiveness. Gonçalves et al. reported lung cancer cells seeded in matrices with low collagen density migrate more in a centre-based computational model, hinting high collagen density inhibits tumour cell migration [30], which is consistent with our finding. However, Kaur et al. showed that HAPLN1 is lost in aged fibroblasts, resulting in a more aligned ECM that promoted metastasis of melanoma cells [17], but our data indicated that HAPLN1 increased the fiber alignment in gastric cancer, which in turn promoted tumour invasion. Provenzano et al. reported that increased stromal collagen density results in increases of tumour formation, tumour invasive phenotype and more metastasis in breast cancer [31]. The inconsistent effects of ECM remodeling on tumour progress in such non-visceral tumours as dermal or mammary neoplasms *versus* visceral tumours, including gastric cancer, may also attribute to the organ-specific functionality of ECM [32–34].

Our present study has some inadequacies. CAFs are abundant and heterogeneous stromal cells in TME [3]. Different functional CAFs subpopulations with distinct phenotypes have been identified to exert specific effects on tumour progression [35]. We will make certain the CAFs subtype responsible for producing HAPLN1 in the future. Furthermore, the phenotypic alterations following HAPLN1 deletion or overexpression in gastrointestinal system remain unknown, albeit several studies have addressed HAPLN1-associated phenotypes [17, 18, 20, 21]. We will pursuit our future research to investigate HAPLN1 regulation in stomach to elucidate its detailed roles in gastric tumourgenesis and tumour progress.

**Fig. 7:**
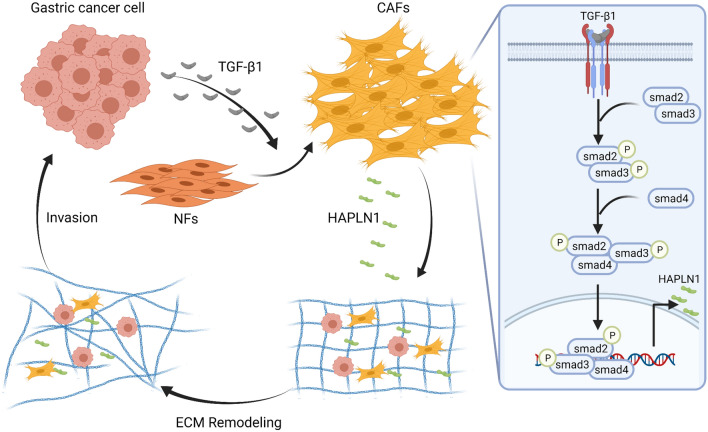
Schematic diagram indicating CAFs-derived HAPLN1 promotes tumour invasion through ECM remodeling in gastric cancer

Collectively, our study demonstrated that gastric cancer cells promote HAPLN1 production in CAFs via activation of TGF-β1/Smad signaling. CAFs-derived HAPLN1 is associated with disease progression and poor prognosis in gastric cancer, and CAFs-derived HAPLN1 enhances gastric cancer cell invasiveness through extracellular matrix remodeling (Fig. 7). Our findings facilitate our understanding of roles of CAFs in the pathogenesis of gastric cancer, which could lead to the development of novel strategies of stroma-based diagnostics, prognostics and therapeutics in cancers.

## Supplementary Information

Below is the link to the electronic supplementary material.

Supplementary file1 (DOCX 28 KB)

Supplementary file2 (DOCX 20 KB)

Supplementary file3 (DOCX 18 KB)

Supplementary file4 (DOCX 32 KB)

Supplementary file5 (DOCX 21 KB)

Supplementary file6 Figure s1. (A) Treatment with the condition medium (CM) of AGS cells or MKN45 cells increased HAPLN1 expression in Hs738 cells with a time-dependent manner. (B) TGF-β1 levels in human gastric cancer cell lines, including HGC27, MKN45, AGS and MGC803 cells, and was higher than those in primary CAFs, NFs and Hs738 cell. (C) Anti-TGF-β1 neutralizing monoclonal antibody could inhibit the effects of gastric cancer cell CM on HAPLN1 expression in Hs738 cells, and decreased HAPLN1 production of Hs738 cells induced by tumour cell CM (D). (E) TGF-β1 treatment up-regulated the expression of FAP, α-SMA, HAPLN1, Smad2/3, Smad2, p-Smad2, Smad3 and p-Smad3 in Hs738 cells. (F) Treatment with gastric cancer cell conditioned medium also increased Smad2/3 expression in Hs738 cells, and anti-TGF-β1 could inhibit these effects accordingly (G). (H) S8144 could down-regulate both HAPLN1 and Smad2/3 expression in Hs738 cells with a time-dependent manner. (* P<0.05, ** P<0.01, *** P<0.001 vs con or 0h) (TIF 10234 KB)

Supplementary file7 Figure s2. (A) CAFs supernatant promoted cell migration of MKN45 in wound-healing assay. (B) CAFs supernatant promoted MKN45 cell invasion using Transwell assay. (C) HAPLN1 expression in CAFs was inhibited by shRNA #1 or #2. (D and E) CAFs supernatant promoted cell migration and invasion of MKN45; however, treatment with CAFs-sh HAPLN1 supernatant inhibited these effects. MKN45 cell migration or invasion was recovered by co-culture with rHAPLN1. (F and G) 3D spheroid cell invasion assay showed that CAFs increased MKN45 cell invasion, while HAPLN1 knockdown dramatically reduced the CAFs-mediated promotion of invasive ability of MKN45 cells. Addition of rHAPLN1 could recover these effects. (* P<0.05, ** P<0.01, *** P<0.001) (TIF 13761 KB)

Supplementary file8 Figure s3. (A) HAPLN1 expression in CAFs was inhibited by shRNA. (B) Nude mouse studies indicated that the tumour volume in MKN45/CAFs-sh HAPLN1 group was smaller than that in MKN45/CAFs group, and rHAPLN1 counterbalanced this effect significantly. (C) Assays with these tumours in mice confirmed the HAPLN1 levels in different groups. (D) HAPLN1 knockdown increased the number, density, width and length of fibers in ECM, and reduced the fiber alignment significantly, while rHAPLN1 treatment could counteract these effects of HAPLN1 knockdown on ECM remarkably. (E) Stromal HAPLN1 expression was increased with Grade 1 to Grade 3 in human gastric cancer significantly. (* P<0.05, ** P<0.01, *** P<0.001) (sh NC: null control of shRNA) (TIF 529 KB)

## Data Availability

The datasets used and/or analyzed during the current study are available from the corresponding author upon reasonable request.
