# Supplementary material for: Cancer-associated fibroblasts-derived HAPLN1 promotes tumour invasion through extracellular matrix remodeling in gastric cancer
Source: Gastric Cancer. 2021 Nov 1;25(2):346–59. doi: 10.1007/s10120-021-01259-5 (PMC8882084; doi:10.1007/s10120-021-01259-5)
Supplement: Supplementary file 1 — Supplementary file1 (DOCX 28 KB) [file 10120_2021_1259_MOESM1_ESM.docx]

**Supplementary Materials and Methods**

**Cell culture, wound healing assay and Transwell invasion assay**

Human gastric adenocarcinoma cell lines, AGS (ATCC, VA, USA) and MGC803 (CBTCCCAS, Shanghai, China), SGC7901 (CBTCCCAS, Shanghai, China), HGC27 (CBTCCCAS, Shanghai, China), MKN45 (CBTCCCAS, Shanghai, China) and human stomach fibroblast line Hs738 (ATCC, VA, USA) were cultured in the complete DMEM (ATCC, VA, USA) supplemented with 10% fetal bovine serum (Hyclone, Logan, UT, USA). Isolation and culture of primary gastric cancer CAFs and primary stomach NFs in corresponding non-cancerous tissues were performed according to our previous report [4, 5] and referring the newly-published protocol of Yasuda et al [12]. Briefly, fresh samples were washed with serum-free DMEM, cut into small pieces, and were transferred to a 0.15% collagenase IV solution, followed by incubation at 37 °C for 40 min. Digested cells were filtered through a 40-mm cell strainer (Milex-GP) and centrifuged at 1500 rpm for 10 min. The single-cell suspension was incubated in a Fibroblast Medium Kit (Cat. No. P60108, Innoprot) for 24 h, allowing fibroblasts to attach on culture plates. Unattached cells were removed after 24 h incubation, and the adherent cells were further cultivated for experiments. Cultured CAFs and NFs less than five passages were used for our experiments. Primary CAFs and the corresponding NFs were cultured in the complete RPMI1640 (GIBCO, VA, USA) supplemented with 10% fetal bovine serum (Hyclone, Logan, UT, USA). Wound healing assay and Transwell invasion assay were performed according to our previous report [5].

**Real-time quantitative PCR (qPCR)**

Real-time quantitative PCR (qPCR) was performed to detect HAPLN1 mRNA levels in human gastric cancer tissues. The primer for HAPLN1 was sense CAGACCTCACTCTGGAAGATTATG, and anti-sense GGGAATACCAGACCTTGTAAGT.

**Enzyme-linked immunosorbent assay (ELISA)**

ELISA was used to detect HAPLN1 in the cell supernatants and in the patient serums. A rabbit monoclonal anti-HAPLN1 antibody (Abcam, Cambridge, UK) was used. Each experiment was repeated at least three times.

**Western blotting assay**

Protein expression levels of the indicated molecules were detected using the western blotting assay. The monoclonal antibodies used for the analyses were as following: rabbit anti-HAPLN1 antibody, rabbit anti-transforming growth factor-β1 (TGF-β1) antibody (Abcam, Cambridge, UK), rabbit anti-fibroblast activated protein (FAP) antibody, rabbit anti-α-smooth muscle actin (α-SMA) antibody, rabbit anti-Smad2 antibody, rabbit anti-phosphorylated Smad2 (anti-p-Smad2) antibody, rabbit anti-p-Smad3 antibody, rabbit anti-Smad2/3 antibody, rabbit anti-Smad4 antibody (Cell Signaling Technology, MA, USA). Relative levels were quantified and normalized with β-actin or GAPDH in the same sample with density analysis.

**Immunofluorescence**

The section thickness of paraffin-embedded samples is 4 mm. In 0.01M citrate buffer, antigen was extracted in a pressure cooker within 20 min. Then, in PBS containing 10% bovine serum albumin, the sections were blocked at room temperature for 2 h. After blocking, samples were incubated with primary antibodies specific for mouse anti-a-SMA (1:100) (Cell Signaling Technology, MA, USA), rabbit monoclonal anti-HAPLN1 (1:250) (Abcam, Cambridge, UK) and mouse monoclonal anti-TGF-β1 antibody (1:200) (Affinity, Cincinnati, OH, USA) overnight at 4℃. Fluorescent secondary antibody was carried out for 1 h at room temperature. Cell nuclei were counterstained with DAPI (Sigma-Aldrich, MO, USA). Images were acquired on a Zeiss LSM510 confocal microscope (Oberkochen, Germany).

**Spheroid cell invasion assay**

Spheroid cell invasion assay was carried out using the 96 Well 3D Spheroid BME Cell Invasion Reagent Kit (Trevigen, MD, USA). The procedures of cell culture were according to the manufacturer’s recommendation and our previous report [5].
