# Supplementary material for: Cancer-associated fibroblasts-derived HAPLN1 promotes tumour invasion through extracellular matrix remodeling in gastric cancer
Source: Gastric Cancer. 2021 Nov 1;25(2):346–59. doi: 10.1007/s10120-021-01259-5 (PMC8882084; doi:10.1007/s10120-021-01259-5)
Supplement: Supplementary file 2 — Supplementary file2 (DOCX 20 KB) [file 10120_2021_1259_MOESM2_ESM.docx]

**Table s1. Three primer pairs of HAPLN1 used in ChIP analyses**

| pair #1 | sense | CCTGTGCCCATCTGGTTCTAA |
| --- | --- | --- |
|  | anti-sense | TCTGTGGAATGATCTGCGGG |
|  |  |  |
| pair #2 | sense | CCCTCGCATTCCTCGTTCTAT |
|  | anti-sense | TGGATGGCAGAGGTCCTGAT |
|  |  |  |
| pair #3 | sense | CCTGTGCCCATCTGGTTCTAAT |
|  | anti-sense | GGTCTGTCCAGAAGGGATGG |
