# Supplementary material for: Cancer-associated fibroblasts-derived HAPLN1 promotes tumour invasion through extracellular matrix remodeling in gastric cancer
Source: Gastric Cancer. 2021 Nov 1;25(2):346–59. doi: 10.1007/s10120-021-01259-5 (PMC8882084; doi:10.1007/s10120-021-01259-5)
Supplement: Supplementary file 3 — Supplementary file3 (DOCX 18 KB) [file 10120_2021_1259_MOESM3_ESM.docx]

**Table s2. Two HAPLN1 shRNAs used in HAPLN1 silencing**

| shRNA #1 | GCCAAACTGTTTAAGCTGTAT |
| --- | --- |
| shRNA #2 | GACCAAGCTAACTTCGGATTA |
