# Supplementary material for: Cancer-associated fibroblasts-derived HAPLN1 promotes tumour invasion through extracellular matrix remodeling in gastric cancer
Source: Gastric Cancer. 2021 Nov 1;25(2):346–59. doi: 10.1007/s10120-021-01259-5 (PMC8882084; doi:10.1007/s10120-021-01259-5)
Supplement: Supplementary file 4 — Supplementary file4 (DOCX 32 KB) [file 10120_2021_1259_MOESM4_ESM.docx]

**Table s3. The clinical relevance of HAPLN1 expression in gastric cancer**

| Variable | N  (155) | HAPLN1 expression | | *P* value |
| --- | --- | --- | --- | --- |
|  |  | Low (75) | High (80) |  |
| Age (y) |  |  |  | >0.9999 |
| ≤ 60 | 61 | 30 | 31 |  |
| > 60 | 94 | 45 | 49 |  |
| Gender |  |  |  | 0.2027 |
| Male | 114 | 59 | 55 |  |
| Female | 41 | 16 | 25 |  |
| Tumor differentiation |  |  |  | 0.0146 |
| Grade Ⅰ+ Grade Ⅱ | 41 | 27 | 14 |  |
| Grade Ⅲ | 90 | 38 | 52 |  |
| Depth of tumor invasion |  |  |  | <0.0001 |
| T1~2 | 48 | 39 | 9 |  |
| T3~4 | 107 | 36 | 71 |  |
| Lymph node metastasis |  |  |  | 0.0006 |
| N0~1 | 63 | 41 | 22 |  |
| N2~3 | 92 | 34 | 58 |  |
| Distant metastasis |  |  |  | 0.4973 |
| M0 | 153 | 75 | 78 |  |
| M1 | 2 | 0 | 2 |  |
| TNM stage |  |  |  |  |
| Ⅰ~Ⅱ | 71 | 43 | 28 | 0.0063 |
| Ⅲ~Ⅳ | 84 | 32 | 52 |  |
