# Supplementary material for: Cancer-associated fibroblasts-derived HAPLN1 promotes tumour invasion through extracellular matrix remodeling in gastric cancer
Source: Gastric Cancer. 2021 Nov 1;25(2):346–59. doi: 10.1007/s10120-021-01259-5 (PMC8882084; doi:10.1007/s10120-021-01259-5)
Supplement: Supplementary file 5 — Supplementary file5 (DOCX 21 KB) [file 10120_2021_1259_MOESM5_ESM.docx]

**Table s4. The correlation of HAPLN1 expression and TGF-β1 expression in gastric cancer tissues.**

|  | | HAPLN1 | | *P* value |
| --- | --- | --- | --- | --- |
|  |  | Low | High |  |
| TGF-β1 | Low | 8 | 11 | 0.0165 |
|  | High | 12 | 29 |  |
