# Supplementary figures and images for: Cancer-associated fibroblasts-derived HAPLN1 promotes tumour invasion through extracellular matrix remodeling in gastric cancer
Source: Gastric Cancer. 2021 Nov 1;25(2):346–59. doi: 10.1007/s10120-021-01259-5 (PMC8882084; doi:10.1007/s10120-021-01259-5)

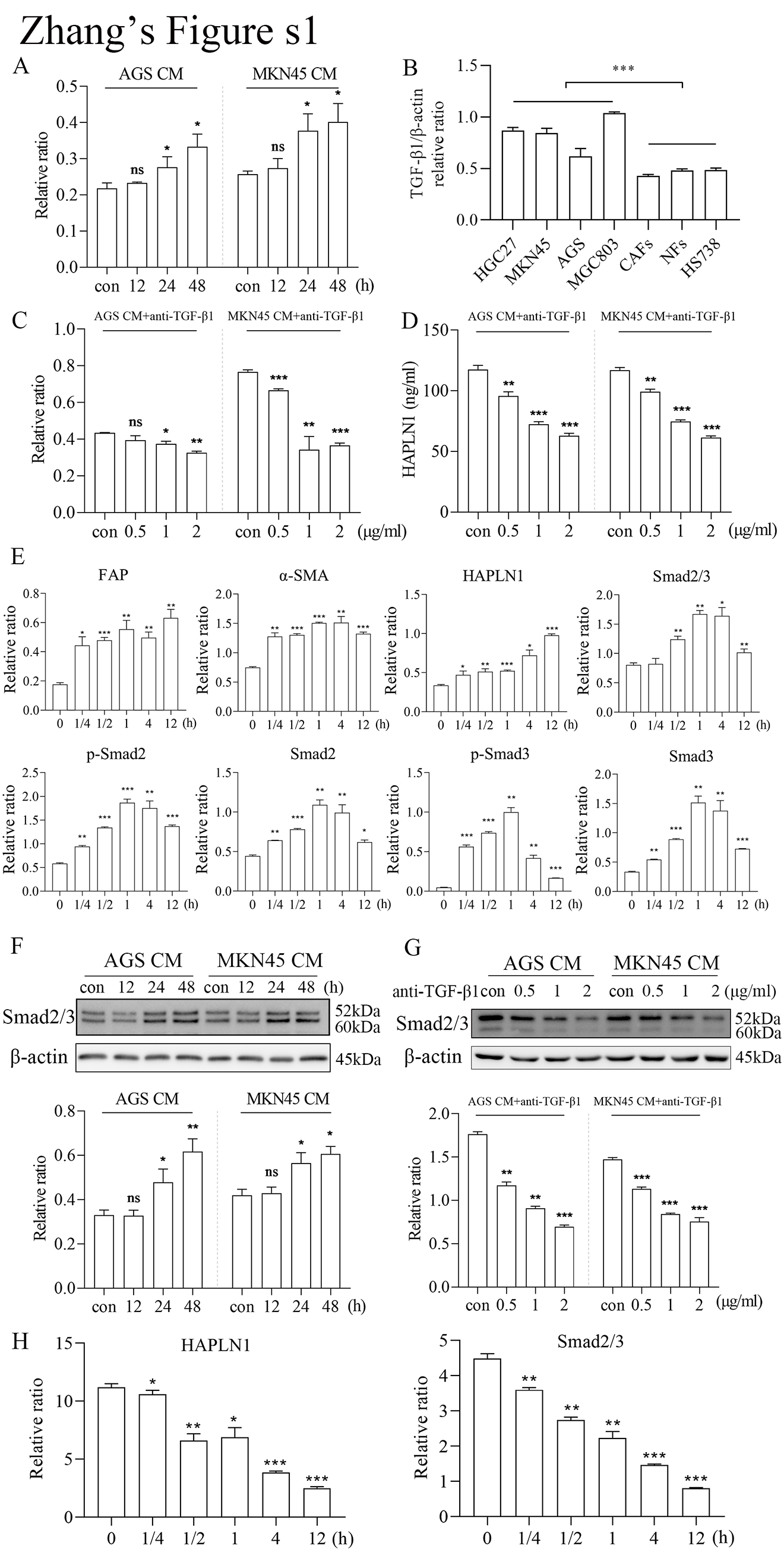

Supplement: Supplementary file 6 — Supplementary file6 Figure s1. (A) Treatment with the condition medium (CM) of AGS cells or MKN45 cells increased HAPLN1 expression in Hs738 cells with a time-dependent manner. (B) TGF-β1 levels in human gastric cancer cell lines, including HGC27, MKN45, AGS and MGC803 cells, and was higher than those in primary CAFs, NFs and Hs738 cell. (C) Anti-TGF-β1 neutralizing monoclonal antibody could inhibit the effects of gastric cancer cell CM on HAPLN1 expression in Hs738 cells, and decreased HAPLN1 production of Hs738 cells induced by tumour cell CM (D). (E) TGF-β1 treatment up-regulated the expression of FAP, α-SMA, HAPLN1, Smad2/3, Smad2, p-Smad2, Smad3 and p-Smad3 in Hs738 cells. (F) Treatment with gastric cancer cell conditioned medium also increased Smad2/3 expression in Hs738 cells, and anti-TGF-β1 could inhibit these effects accordingly (G). (H) S8144 could down-regulate both HAPLN1 and Smad2/3 expression in Hs738 cells with a time-dependent manner. (* P<0.05, ** P<0.01, *** P<0.001 vs con or 0h) (TIF 10234 KB) [file 10120_2021_1259_MOESM6_ESM.tif]

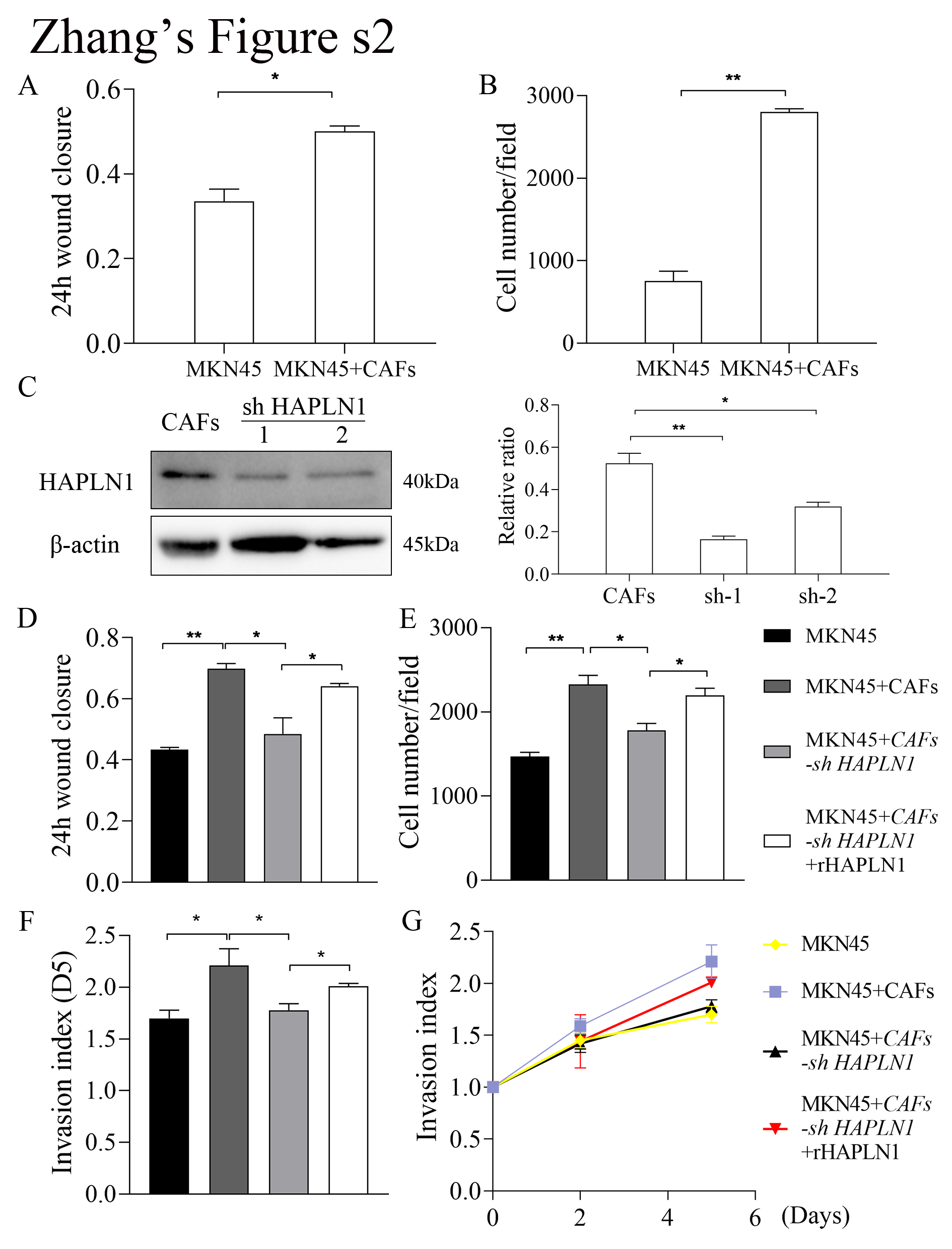

Supplement: Supplementary file 7 — Supplementary file7 Figure s2. (A) CAFs supernatant promoted cell migration of MKN45 in wound-healing assay. (B) CAFs supernatant promoted MKN45 cell invasion using Transwell assay. (C) HAPLN1 expression in CAFs was inhibited by shRNA #1 or #2. (D and E) CAFs supernatant promoted cell migration and invasion of MKN45; however, treatment with CAFs-sh HAPLN1 supernatant inhibited these effects. MKN45 cell migration or invasion was recovered by co-culture with rHAPLN1. (F and G) 3D spheroid cell invasion assay showed that CAFs increased MKN45 cell invasion, while HAPLN1 knockdown dramatically reduced the CAFs-mediated promotion of invasive ability of MKN45 cells. Addition of rHAPLN1 could recover these effects. (* P<0.05, ** P<0.01, *** P<0.001) (TIF 13761 KB) [file 10120_2021_1259_MOESM7_ESM.tif]

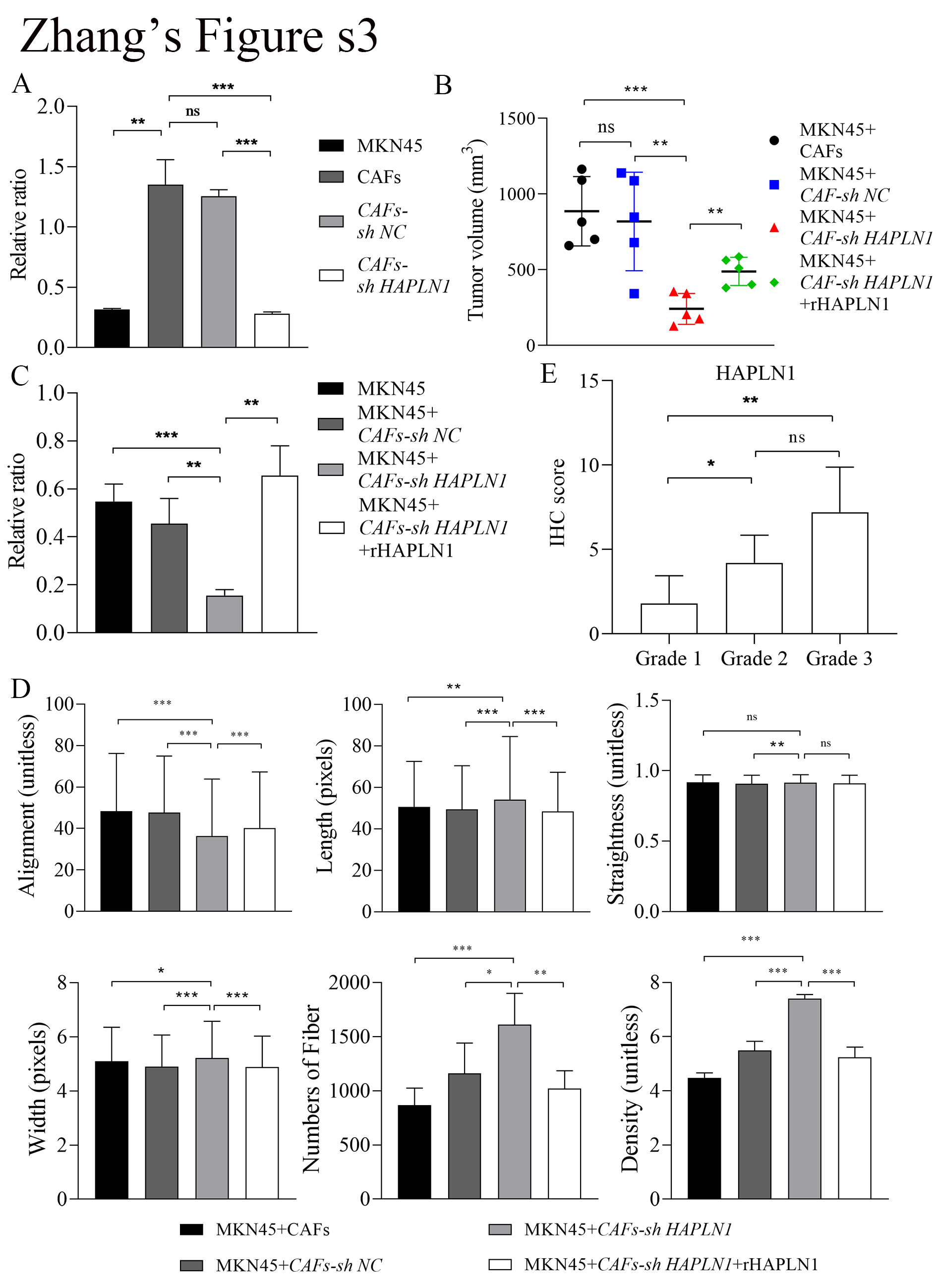

Supplement: Supplementary file 8 — Supplementary file8 Figure s3. (A) HAPLN1 expression in CAFs was inhibited by shRNA. (B) Nude mouse studies indicated that the tumour volume in MKN45/CAFs-sh HAPLN1 group was smaller than that in MKN45/CAFs group, and rHAPLN1 counterbalanced this effect significantly. (C) Assays with these tumours in mice confirmed the HAPLN1 levels in different groups. (D) HAPLN1 knockdown increased the number, density, width and length of fibers in ECM, and reduced the fiber alignment significantly, while rHAPLN1 treatment could counteract these effects of HAPLN1 knockdown on ECM remarkably. (E) Stromal HAPLN1 expression was increased with Grade 1 to Grade 3 in human gastric cancer significantly. (* P<0.05, ** P<0.01, *** P<0.001) (sh NC: null control of shRNA) (TIF 529 KB) [file 10120_2021_1259_MOESM8_ESM.tif]
